# Use of Tranexamic acid is a cost effective method in preventing blood loss during and after total knee replacement

**DOI:** 10.1186/1749-799X-6-22

**Published:** 2011-05-21

**Authors:** Yasir J Sepah, Masood Umer, Tashfeen Ahmad, Faria Nasim, Muhammad Umer Chaudhry, Muhammad Umar

**Affiliations:** 1 Aga Khan University Medical College, Karachi-74800, Pakistan; 2 Associate Professor Department of Surgery (Orthopedics) Aga Khan University Hospital, Karachi-74800, Pakistan; 3 Assistant Professor Department of Surgery (Orthopedics) Aga Khan University Hospital, Karachi-74800, Pakistan; 4 Professor Department of Surgery (Orthopedics) Aga Khan University Hospital, Karachi-74800, Pakistan

## Abstract

**Background & Purpose:**

Allogenic blood transfusion in elective orthopaedic surgery is best avoided owing to its associated risks. Total knee replacement often requires blood transfusion, more so when bilateral surgery is performed. Many strategies are currently being employed to reduce the amount of peri-operative allogenic transfusions. Anti-fibrinolytic compounds such as aminocaproic acid and tranexamic acid have been used systemically in perioperative settings with promising results. This study aimed to evaluate the effectiveness of tranexamic acid in reducing allogenic blood transfusion in total knee replacement surgery.

**Methodology:**

This was a retrospective cohort study conducted on patients undergoing total knee replacement during the time period November 2005 to November 2008. Study population was 99 patients, of which 70 underwent unilateral and 29 bilateral knee replacement. Forty-seven patients with 62 (49.5%) knees (group-I) had received tranexamic acid (by surgeon preference) while the remaining fifty-two patients with 66 (51.5%) knees (group-II) had did not received any tranexamic acid either pre- or post-operatively.

**Results:**

The mean drop in the post-operative haemoglobin concentration in Group-II for unilateral and bilateral cases was 1.79 gm/dl and 2.21 gm/dl, with a mean post-operative drainage of 1828 ml (unilateral) and 2695 ml (bilateral). In comparison, the mean drop in the post-op haemoglobin in Group-I was 1.49 gm/dl (unilateral) and 1.94 gm/dl (bilateral), with a mean drainage of 826 ml (unilateral) and 1288 ml (bilateral) (p-value < 0.001).

**Interpretation:**

Tranexamic acid is effective in reducing post-operative drainage and requirement of blood transfusion after knee replacement.

## Introduction

Total knee replacement is a frequently done procedure in modern day practice of any Orthopedics unit. Limiting blood loss both postoperatively and intra-operatively presents a challenge to the surgeon. Postoperatively, blood continues to ooze from the cut ends of bone, the open intra-medullary canal and the raw, dissected soft tissues. This can amount to significant bleeding with figures ranging from 600 - 1500 ml [1-7]. As this procedure is performed under tourniquet control, there is an associated increase in localized fibrinolysis, which contributes to two events. Firstly, it decreases the risk of venous thromboembolism and secondly it may aggravate post-operative haemorrhage [8-10]. The problem of excessive blood loss is further highlighted in cases of simultaneous bilateral total knee replacement where blood loss is usually twice that of a unilateral knee replacement and the number of allogenic blood units transfused can be as high as three to four per person [11,12]. Risks associated with allogenic blood transfusion are numerous and well documented. Of these the most important are blood borne infections, immunological reactions and cost incurred in producing a unit of red cells [13-15].

Tranexamic acid is an antifibrinolytic agent, which effectively blocks this fibrinolytic activity, thus causing a marked reduction in post-operative bleeding. It works by blocking the lysine binding sites of plasminogen and prevents the degradation of fibrin. It has been previously used quite successfully in urological, gynecological and thoracic surgical procedures in order to reduce post-operative blood loss [16-19]. The use of tranexamic acid in orthopedic surgery has also shown promising results. It radically reduced both blood loss and the amount of allogenic transfusions needed postoperatively [9,20-22]. Considering the high risks associated with the use of allogenic blood, we think that this drug can be very beneficial to patients undergoing both unilateral and simultaneous bilateral total knee replacements.

■ The aim of this study was to determine: if the use of tranexamic acid reduces perioperative blood loss and need for allogenic blood transfusion in patients undergoing total knee replacement

■ Any untoward effects with the use of this drug in our population

## Materials and methods

All patients having undergone total knee replacement at our hospital between November 2005 to November 2008 were included in the study sample.

A total of 99 patients with 128 knee joints were included in the study. Patients from Group I received one gram of IV tranexamic acid before inflation of the tourniquet and 1 g after deflation of tourniquet. Sixty-six patients (66.4%) underwent unilateral and 23 patients (24.6%) had bilateral procedures.

All patients with no known bleeding disorders who under-went TKR were included in our study. All patients were given routine DVT prophylaxis with Injection Enoxaparin 40 mg subcutaneous once a day. Anaesthesia was standardized and all patients received epidural anaesthesia according to standard practice. Patients receiving chronic anticoagulants were excluded from the study. Haemoglobin was measured preoperatively, one hour postoperatively and at 72 hours postoperatively. The same surgical team performed all procedures and the same implant (IB-II - Zimmer, Warsaw, IN) was used in all patients. Patellar replacement was performed in all cases and all components were cemented.

*The established practice of transfusion in our unit is that patients are transfused if:*

1. Postoperative Hb is < 7 mg/dl in patients with no coronary heart disease, or < 9 mg/dl in patients who have coronary heart disease

2. Physiological signs of inadequate oxygenation such as hemodynamic instability or symptoms of myocardial ischemia occur

3. Drainage of more than 1 liter of blood in the first 24 hours

47 patients with 32 (68%) undergoing a unilateral and 15 (32%) undergoing simultaneous bilateral total knee replacement had received tranexamic acid and these were labelled as Group-I. Data form Group-I was collected retrospectively by chart review and then compared with that of Group-II which was a historical control group. Group-II underwent the same procedure of either a unilateral or simultaneous bilateral total knee replacements, but did not receive tranexamic acid and also did not undergo any other procedure to reduce post-operative bleeding. Patients in both groups were age and disease-matched. There were 52 patients in Group-II with 38 (76%) undergoing a unilateral and 14 (24%) undergoing a simultaneous bilateral knee replacement procedure.

Student t-test was used to compare the means via SPSS 13.

## Results

**Figure 1:**
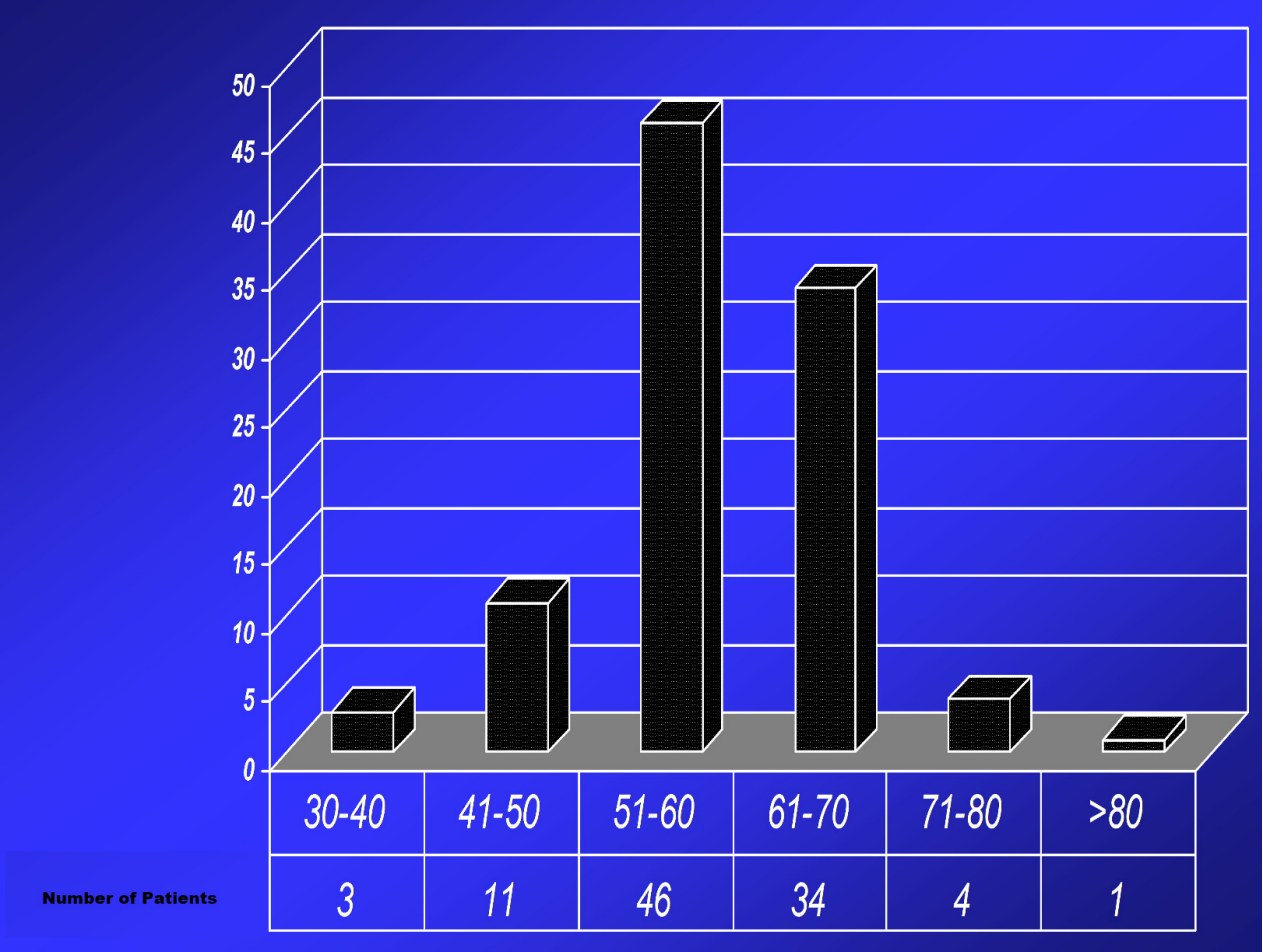
**Age groups**.

Mean age of our study population was 59 years [Figure 1]. 70% were females and 30% were male patients. Indication for surgery in 71% of the patients was osteoarthritis while in 29% of the patients it was rheumatoid arthritis.

**Figure 2:**
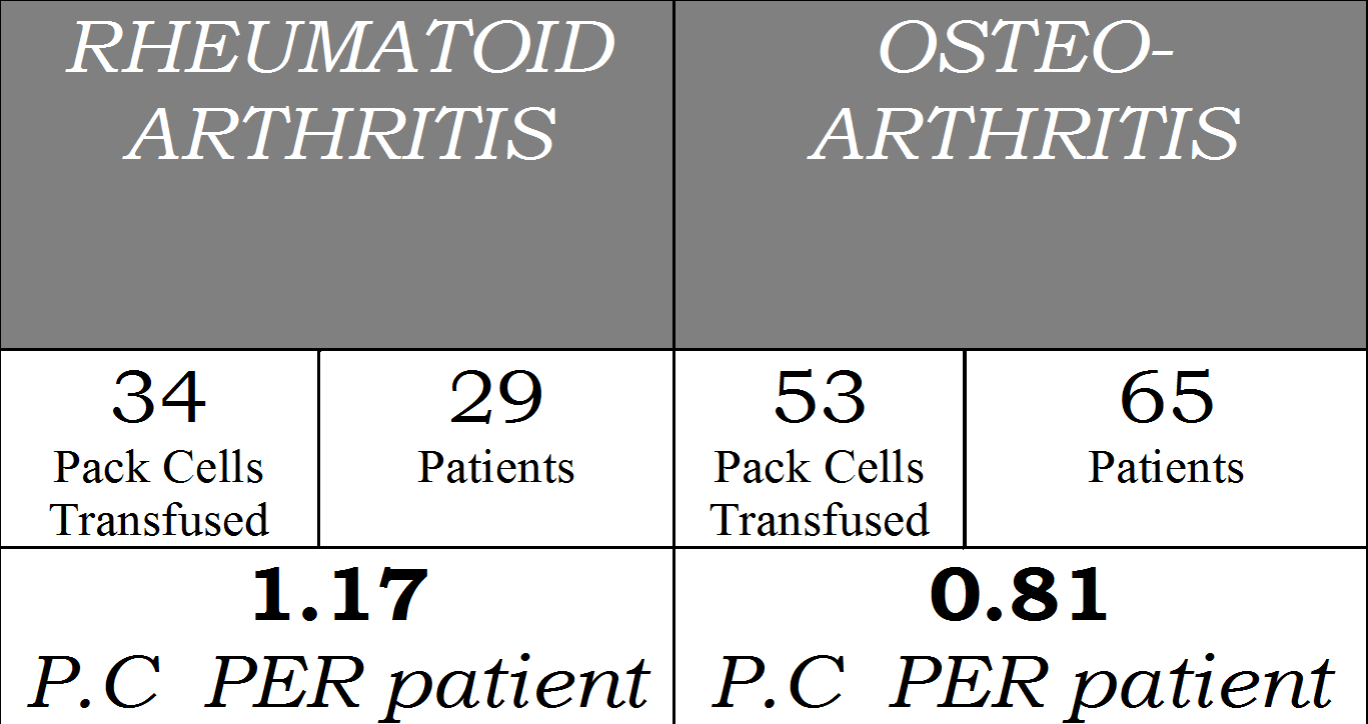
**Comparison of transfusion requirement in Osteoarthritis & Rheumatoid arthritis**.

The mean drop in the post-operative haemoglobin concentration unilateral surgery was 1.49 gm/dl in group Ii and 1.79 gm/dl in group II, a difference of 17%. for bilateral surgery, the drop was 1.94 gm/dl and 2.21 gm/dl respectively, a difference of 12%. mean postoperative blood drainage in unilateral surgery was 826 ml and 1828 ml respectively, a difference of 55%, while in bilateral surgery it was 1288 ml and 2695 ml respectively, a difference of 52%. Mean drop in the post-operative haemoglobin concentration and mean post-operative drainage of both Group-I and Group-II is given in Table 1. Thirty-nine (75%) out of 52 patients in this Group-II required transfusion. Thirty-two (61.53%) patients required one or two units of packed red cells and seven (13.46%) patients required more than two units of transfusion in this group. In comparison, only 6 (12.76%) out of the 47 patients in Group-I required blood transfusion and remaining 22 (88.24%) had not required any transfusion. This difference between the two groups in the number of units of blood transfused is statistically significant (p-value < 0.001). A statistically significant (p-value < 0.01) difference in transfusion requirement for patients with Osteoarthritis and Rheumatoid Arthritis was also noted [Figure 2]. No untoward side effect of tranexamic acid was noted in our patients.

**Table 1:** Summary of results

	*Mean Post-Operative Drainage*		*Mean Drop in Post-Operative HB*		*Mean number of Packed Cells Transfused*		*Number of Patients requiring Transfusion*
	*Unilateral*	*Bilateral*	*Unilateral*	*Bilateral*	*Unilateral*	*Bilateral*	
Group-I (N = 47)	826 ml	1288 ml	1.49 g/dl	1.94 g/dl	0.12	0.9	6
Group-II (N = 52)	1828 ml	2695 ml	1.79 g/dl	2.21 g/dl	1.24	2.6	35
p value	< 0.001	< 0.001	0.0005	< 0.0005	0.005	0.043	

## Discussion

Knowing all the risks and morbidity associated with allogenic blood transfusion, a surgeon always looks for ways and means whereby allogenic blood transfusion can be avoided in surgical patients. The most noticeable, and extensively explored options available are preoperative blood donation (PAD), acute normovolemic hemodilution (ANH), perioperative red cell salvage (PCS) and certain anaesthetic techniques (deliberate hypotension, normothermia) [23]. Certain pharmacological interventions that have been used with success are Recombinant Human Erythropoietin, tranexamic acid and Aprotinin. However, none of these agents are without complications [table 2] and the most important factors to consider in the developing world are the availability and cost effectiveness of these strategies.

**Table 2:** Different methods of blood conservation and their complications.

Alternatives used to avoid allogenic blood transfusions and their disadvantages	
**Preoperative Blood Donation (PAD)**	• Cardiac, Vasovagal *(Risk Factors: Younger Age, Lower weight, 1*^*St *^*time donation*) [34] • 12 times increase in Anginal and Vasovagal complications *(Risk Factor: 1*^*st *^*time donation) *[35] • Overall increase frequency of transfusion *(Risk Factor: Lower Preoperative Hematocrit*) [36,37] • Not Cost Effective in Orthopaedic Procedures [38]*(More expensive to produce one unit of autologous blood, Cost also incurred in disposal of more than half of the blood discarded which is not used)*

**Acute Normovolemic Hemodilution (ANH)**	Not effective in Orthopedic Procedures *(data termed inconclusive) *[39]

**Perioperative red cell salvage (PCS)**	Cost effectiveness of the postoperative blood collection devices was challenged *(1*^*st *^*six hour collection would cost 31-35 million dollars) *[40]

**Deliberate Hypotension DH)**	Persistent hypotension, Reactionary haemorrhage, Cardiac Ischemic Injury, Ischemic Optic neuritis [41-43]

**Recombinant Human Erythropoietin (RHE)**	Routine use not justified due to high cost [44]

**Tranexamic acid**	Very effective [9,20]

**Aprotinin**	• Low dose not effective in orthopedic procedure [45] • Evidence has been published to suggest an increase in renal events in patients given aprotinin when compared to those where tranexamic acid was used [46]

Tranexamic acid, by way of its anti-fibrinolytic action, prevents clot breakdown and a consequent re-bleed. Our results demonstrate significant reduction in blood loss with the use of tranexamic acid. Other studies have also had similar results [20,22]. A meta analysis which looked at double blinded randomized controlled trial also found that tranexamic acid was useful in reducing blood loss in major orthopedic procedures [24].

No adverse effects were seen in our population with the use of tranexamic acid. Although side effects have been reported in other large scale studies but none of them were serious enough to warrant disuse of the drug [25,26].

In South Asia, apart from poverty, low literacy, social factors that result in the inability of women to negotiate safe sex, intravenous drug use and unsafe transfusion is regarded as one of the most important factors that influence transmission of infection [27,28]. High frequency of viremia due to transfusion-transmitted virus was observed in most of the study populations from third world countries, with values ranging from 16 percent in Pakistan to 83 percent in Gambia [29]

A large scale study [30] in Pakistan has shown that the screening coverage on the average has been 77.42% for HIV and 86.84% for HBV. The prevalence of HIV is 0.001% and of HBV is 2.259% [30]. The probability of receiving an infective unit P(R) per 10000 donations is 0.023 for HIV and 29.72 for HBV. The probability of transmitting infection P (I) per 10000 donations is 0.021 for HIV and 26.75 for HBV. The spreading index for both viral infections combined is 26.75 per 10000 donations. Although 80% of joint replacement procedures take place in the United States and Europe, South Asia is not far behind with an estimated 40-50 thousand joint replacement procedures already done yearly in India alone [31]. Number of knees replaced annually in Pakistan is estimated to be 1500-2000 [32].

The cost of one unit of red cells is estimated 120 pounds [14] in Britain while it costs 19.20 British Pounds in Pakistan [33]. The regimen of tranexamic acid that was administered in our study population costs 3.75 Pounds. These figures reflect that if one is able to decrease the requirement of blood by even one unit per patient the cumulative effect will be a decrease of burden on the health care system. Countries where individuals pay for their own health care and there is no third party plan (health insurance companies) involved can benefit from adopting such cost effective measures.

Although we conducted a retrospective analysis of a relatively small number patients and the possibility of the results being affected by recall bias due to historical controls cannot be ruled out it does provide the basis for conducting larger scale prospective randomized studies in order to determine the efficacy of tranxemic acid in reducing perioperative blood loss.

We believe that the use of tranexamic acid in TKR surgery is a low cost option in reducing the requirement of allogenic blood transfusion.

## Competing interests

The authors declare that they have no competing interests.

## Authors' contributions

YJS did the overall supervision and participated in the conception of the idea, preparation of the questionnaire and protocol, collection of data and writing the manuscript. MU was involved in the overall supervision, preparation of the questionnaire and collection and analysis of data. TA was involved in the study design, analysis and was involved in critically reviewing the manuscript. FA and MUC participated in the designed the study and participated in the preparation of the protocol and data collection. MU participated in overall supervision and critically reviewed the manuscript. All authors read and approved the final manuscript.
